# Lateral pelvic lymph node dissection (LPLND) in the treatment of rectal cancer: current practice and evolving approaches in India

**DOI:** 10.1007/s10151-024-03081-4

**Published:** 2025-01-23

**Authors:** Ankit Sharma, Subhathira Manohkaran, Avanish Saklani

**Affiliations:** Colorectal Division, Department of Surgical Oncology, Tata Memorial Hospital, Mumbai, India

**Keywords:** Pelvic lymph node, Rectal cancer, Total neoadjuvant therapy

## Abstract

**Background:**

The introduction of total mesorectal excision improved locoregional control for rectal adenocarcinoma significantly. Standardisation of the technique of LPLND is lacking in literature.

**Methods:**

We describe the current practices of case selection and technical details of lateral lymph node dissection in rectal cancer. We also describe the approach when post neo-adjuvant fibrosis renders standard resection unsafe.

**Results:**

Careful case selection and standardisation of the lateral lymph node dissection technique is important to ensure an oncologically sound and surgically procedure . Step-by-step procedures of LPLND are described in this article, and a video is demonstrated.

**Conclusions:**

Standardisation of the techniques of lateral lymph node dissection is essential. The procedure has a definite learning curve, requiring considerable expertise to avoid complications and achieve optimal outcomes.

**Supplementary Information:**

The online version contains supplementary material available at 10.1007/s10151-024-03081-4.

## Introduction

Lateral pelvic lymph node dissection (LPLND) plays a crucial role in managing rectal cancer, particularly in cases where lymph nodes persist after chemoradiation. In current practice in India, LPLND is selectively performed based on criteria established by Ogura and colleagues, focusing on lymph node size as determined by MRI scans [1]. According to the requirements outlined by Ogura, lymph nodes measuring ≥ 7 mm on a baseline MRI and persistent lymph nodes of 6 mm for obturator nodes and 4 mm for internal iliac nodes are indicated for LPLND. This selective approach aims to balance the need for aggressive cancer control with the desire to avoid unnecessary complications from extensive lymph node dissection.

In patients with locally advanced rectal cancer, involvement of lateral pelvic lymph nodes (LPLN) is recognized as a high-risk factor for poor prognosis, along with other indicators such as mesorectal fascia involvement, extramural venous invasion (EMVI), T4 stage, and N2 status. Additionally, the presence of tumour deposits along with EMVI and poorly differentiated tumours may be linked to a higher incidence of LPLN involvement [2, 3].

Historical studies have reported that the incidence of lateral pelvic lymph node (LPLN) involvement in rectal cancers located below the peritoneal reflection (Rb rectal cancer, according to Japanese classification) for cT3 and higher stages is approximately 20% [1, 4]. In contrast, the incidence of LPLN involvement in early-stage rectal cancer, such as T2N0, is relatively low, but significant percentages of lateral recurrence are seen [6]. Research suggests that upfront surgery alone [total mesorectal excision (TME) with/without pelvic node dissection] but without neoadjuvant chemoradiation for enlarged lateral pelvic nodes is associated with high rates of lateral recurrence [5]. A study from Japan reported a lateral recurrence rate of nearly 22.8% in such cases, indicating that surgery alone may not provide adequate control [6]. Consequently, long-course chemoradiotherapy (LCRT) is often recommended, even for early-stage rectal cancer, to mitigate the risk of recurrence.

Neoadjuvant chemoradiotherapy (CCRT) results in nodal regression in approximately 50% of cases, as observed in restaging MRI [7]. However, despite this radiological regression, pathological analysis following lateral pelvic lymph node dissection (LPLND) continues to reveal positive lymph nodes in 25–50% of patients, indicating persistent disease in a substantial proportion of cases [8, 9].

The decision between short- and long-course radiation therapy or even various regimes of total neoadjuvant treatment (TNT) for patients with LPLN involvement has not been definitively resolved. The RAPIDO trial, which included high-risk cases with LPLN involvement, did not separately address treatment strategies or outcomes for this subset of patients [10]. To date, no major clinical trials have specifically focused on this issue. Data from various centers, including those in India, suggest that both treatment approaches may offer comparable outcomes [11, 12]. Notably, the OPRA trial, which primarily included low-risk rectal cancers, reported a lateral recurrence rate of just 3.5% [13].

At some cancer treatment centres in India, the use of a simultaneous integrated boost (SIB) with long-course chemoradiation has shown promising results [14]. This approach aims to intensify the radiation dose to the affected lymph nodes while sparing surrounding tissues, potentially improving response rates. The use of SIB has reduced the need for LPLND from 50 to 30%, highlighting its effectiveness [11, 12, 14]. Similar experiences have been reported with the combination of SIB and short-course radiation therapy [14]. However, performing LPLND after an SIB boost presents significant surgical challenges, particularly for less experienced surgeons, as the boosted radiation can make the tissue more difficult to operate on.

Since the internal iliac and obturator lymph nodes are the two primary nodal groups within the lateral pelvic lymph node (LPLN) compartment that may harbour disease in rectal cancer, lateral pelvic lymph node dissection (LPLND) typically targets the resection of these two sites [15]. The external iliac and common iliac nodes, however, are not routinely included in the dissection. External iliac lymph node dissection may have some advantages in high-risk rectal cancers such as melanomas. The nerve-sparing technique is routinely performed. Boundaries for LPLND are defined laterally by the external iliac artery, medially by the inferior hypogastric nerve and pelvic plexus, cranially by the common iliac bifurcation, and caudally by the obturator internus and pelvic floor. This approach aims to preserve critical nerve structures while allowing for effective lymph node dissection. In some high-risk, advanced cases (EMVI contiguous with lateral pelvic node), the S3/4 nerve plexus has to be sacrificed to achieve complete clearance [16].

The most common intraoperative complication during LPLND is bleeding, with rare instances of damage to surrounding structures such as the ureter. Urinary and sexual dysfunction remain significant challenges, particularly in cases of bilateral pelvic lymph node dissection where extended resection is performed. Other common complications include lymphocele formation, which may be asymptomatic or cause pressure-related issues such as hydroureter or strictures [17, 18].

LPLND has a notable learning curve, requiring both technical proficiency and oncological adequacy [19, 20]. Early in the learning curve, there is a higher likelihood of missing the inferior vesical nodes, which can lead to lateral recurrences despite performing LPLND. Salvage surgery for such recurrences is complex, often necessitating terminal ureter resection and reimplantation [16, 19, 20].

### Surgical procedure overview

LPLND is typically performed after resection of the primary rectal tumour, often after stapling the rectumr. In case of abdominoperineal resection (APR) or after intersphincteric resection (ISR) LPLND is performed after completing the dissection down to the pelvic floor. The procedure begins after ligating the vascular pedicle, completing the total mesorectal excision (TME), and fully mobilizing the rectum down to the pelvic floor.

Exposure and initial steps.

1. Distal sigmoid mobilization: To ensure adequate exposure, the distal sigmoid colon is mobilized and clipped to the opposite side of the dissection field. This provides better access to the pelvic sidewall.

Dissection in males.

2. Peritoneal reflection: The peritoneum is dissected over the iliac vessels and reflected, and this is advanced until the vas deferens. The objective here is to visualize the obliterated hypogastric vessel (umbilical artery).
3. Lateral paravesical space creation: The obliterated hypogastric vessel is pulled medially, allowing for dissection along the lateral border, which creates the lateral paravesical space. Laterally, this exposes the external iliac vein, while medially, the dissection extends to the pubic bone. The obliterated hypogastric vessel follows until its origin from the internal iliac artery is seen.
4. Ureter mobilisation: The ureter is identified near the iliac bifurcation, dissected along the ureterohypogastric fascia, and pulled medially.
5. The medial paravesical space is created by opening the plane along the medial border of the umbilical artery, also known as the vesicohypogastric fascial plane. This space is crucial for accessing lymph nodes around the inferior vesical vessels, ("Achilles heel") because of their high risk of metastasis. In this step, we mobilize the medial umbilical artery laterally to expose and dissect the nodes situated on the internal iliac vein. The lymph nodes located between the vesical vessels are then freed and pushed medially. This approach facilitates the dissection of the inferior vesical nodes while safeguarding the ureter.

Dissection in females.

5. Peritoneal cut: The procedure begins by ligating the round ligament and extending the peritoneal incision along the external iliac vessels. The peritoneal edge is then mobilized with the gonadal vessels up to the iliac bifurcation.
6. Ureter mobilisation: The ureter and gonadals are dissected like in male patients along the ureterohypogastric fascia and can be tagged with a clip to either the rectal stump or the sidewall for better exposure during the dissection.

Dissection of obturator group of nodes.

7. External iliac vein exposure: The external iliac vein is defined, and the nodal packet (fibrofatty tissue) is carefully dissected away along the medial border of this vessel and off the floor of the pelvis. The dissection continues more deeply along the vein up to the medial branch, which communicates with the obturator vein (also known as the "vein of sorrow"). The nodes are cleared from the obturator fossa, taking care to preserve or excise the communicating vein.
8. Obturator nerve preservation: The obturator nerve is carefully separated from the lymph nodes using a "split and roll" technique, ensuring the nerve is not damaged. The nodes are then moved medially for en bloc removal with the internal iliac group of nodes.
9. Difficult vascular delineation occurs in certain cases such as anorectal melanoma and more commonly in patients who have undergone radiotherapy with boost therapy. In such cases, we begin our dissection lateral to external iliac vessels. The vessels are fully detached and mobilized away from the psoas muscle to expose the iliac bifurcation and obturator nerve from behind. Special care is taken to preserve the genitofemoral nerve, which runs on the psoas muscle, close to the site where external iliac artery mobilization begins.

Dissection of internal iliac group of nodes.

10. Medial dissection: The dissection extends medially toward the bladder and pelvic floor until the inferior vesical veins are identified. The nodal packet is carefully dissected, and obturator vessels are transected as needed.
11. **Lumbosacral trunk identification**: The dissection proceeds deeper along the proximal portion of the obturator nerve to identify the lumbosacral trunk, which is traced toward the greater sciatic notch. Venous tributaries from the obturator muscle often cross the lumbosacral trunk and must be carefully managed during this step.
12. **Internal iliac artery branches**: The dissection follows the internal iliac artery, with the first branch, the obturator artery, being clipped at its origin if not already addressed. The dissection continues anteriorly along the internal iliac artery branches, such as the pudendal and inferior vesical arteries, as they exit the lateral pelvic wall. Variations in venous drainage may necessitate clipping of the obturator vein, inferior vesical vein, or the common trunk of the inferior vesical and pudendal veins. Small branches feeding into the nodal packet are clipped or ligated while preserving the superior gluteal artery.
13. **Larger nodes**: When larger nodes are located along the internal iliac vessels or inferior vesical nodes, it may be necessary to ligate the internal iliac artery and, in some cases, the vein at their origins, particularly beyond the superior gluteal artery. The distal ends of these vessels are then dissected close to the bladder to ensure complete clearance.
14. **Final nodal packet dissection**: Once the nodal packet is free from the obturator nerve, lumbosacral trunk, and proximal branches of the internal iliac vessels, it remains attached to the bladder via the vesical vessels and endopelvic fascia. These nodal packets, especially larger ones, are often missed in dissection. The packet is carefully separated from the bladder and perivesical fat, with inferior vesical vessels either preserved or sacrificed depending on the case. The final portion of the nodal packet is then detached from the endopelvic fascia and levator muscles to complete the dissection. In cases with larger nodes or when vessels are taken at their origin, the terminal branches of the pudendal artery and vein may need to be sacrificed.
15. **Approach to bilateral nodes**: For bilateral LPLND, the procedure typically begins on the less affected side to ensure the preservation of at least one superior vesical vessel. Failure to preserve these vessels can compromise bladder blood supply, potentially leading to complications such as cystectomy or pelvic exenteration.
16. **Extended en bloc resection in high-risk patients**: In high-risk patients [e.g., those with extramural venous invasion (EMVI), ≥ grade 2 tumours, tumour deposits, and lateral pelvic lymph node involvement], an extended en bloc resection that includes the primary tumour, S3/4 nerve roots, lateral pelvic nodes, and unilateral seminal vesicle may offer the best chance of achieving an R0 resection.
17. **Use of ICG during LPLND:** We do not use ICG during LPLND. Enlarged lateral nodes can have blocked lymphatic channels which might prevent the ICG from penetrating. Second, identification of a positive lymph node does not change the management, since a formal lateral node dissection will still be required. ‘Node picking’ is associated with worse outcomes [21].

## Conclusion

The management of lateral pelvic lymph nodes in rectal cancer continues to evolve, with advances in chemoradiation and the use of targeted radiation techniques such as SIB boost, and the need for LPLND has been reduced. However, LPLND remains an important option, especially in cases of persistent lymph nodes after neoadjuvant therapy. The procedure is technically demanding, requiring considerable expertise to avoid complications and achieve optimal outcomes. Future research and continued data from ongoing studies will further clarify the role of LPLND and help refine treatment strategies for rectal cancer.

https://figshare.com/account/items/27270687/edit

## Supplementary Information

Below is the link to the electronic supplementary material.

Supplementary file1 (MP4 294378 KB)

## Data Availability

No datasets were generated or analysed during the current study.

## References

[1] Ogura A, Konishi T, Beets GL, Cunningham C, Garcia-Aguilar J, Iversen H, Lateral Node Study Consortium (2019) Lateral nodal features on restaging magnetic resonance imaging associated with lateral local recurrence in low rectal cancer after neoadjuvant chemoradiotherapy or radiotherapy. JAMA Surg 154(9):e192172–e192172 31268504 10.1001/jamasurg.2019.2172 PMC6613303

[2] Abe T, Yasui M, Imamura H, Matsuda C, Nishimura J, Haraguchi N, Ohue M (2022) Combination of extramural venous invasion and lateral lymph node size detected with magnetic resonance imaging is a reliable biomarker for lateral lymph node metastasis in patients with rectal cancer. World J Surg Oncol 20:1–9 34986842 10.1186/s12957-021-02464-3 PMC8728915

[3] Lord A, Brown G, Abulafi M, Bateman A, Frankel W, Goldin R, Nagtegaal I (2021) Histopathological diagnosis of tumour deposits in colorectal cancer: a Delphi consensus study. Histopathology 79(2):168–175 33511676 10.1111/his.14344

[4] Ogawa S, Itabashi M, Inoue Y, Ohki T, Bamba Y, Koshino K, Yamamoto M (2021) Lateral pelvic lymph nodes for rectal cancer: a review of diagnosis and management. World J Gastrointest Oncol 13(10):1412 34721774 10.4251/wjgo.v13.i10.1412 PMC8529924

[5] Ogura A, Shiomi A, Yamamoto S, Komori K, Hamamoto H, Manabe S, Uehara K (2024) Prediction model of the risk for lateral local recurrence in locally advanced rectal cancer without enlarged lateral lymph nodes: lessons from a Japanese multicenter pooled analysis of 812 patients. Ann Gastroenterol Surg 8(2):284–292 38455486 10.1002/ags3.12742 PMC10914708

[6] Ishibe A, Watanabe J, Suwa Y, Suzuki S, Nakagawa K, Suwa H, Endo I (2021) Oncological outcomes of lateral lymph node dissection (LLND) for locally advanced rectal cancer: is LLND alone sufficient? Int J Colorect Dis 36:293–301 10.1007/s00384-020-03760-2 32965528

[7] Ogura A, Konishi T, Cunningham C, Garcia-Aguilar J, Iversen H, Toda S, Lateral Node Study Consortium (2019) Neoadjuvant (chemo) radiotherapy with total mesorectal excision only is not sufficient to prevent lateral local recurrence in enlarged nodes: results of the multicenter lateral node study of patients with low cT3/4 rectal cancer. J Clin Oncol 37(1):33–43 30403572 10.1200/JCO.18.00032 PMC6366816

[8] Peacock O, Manisundaram N, Dibrito SR, Kim Y, Hu CY, Bednarski BK, Chang GJ (2022) Magnetic resonance imaging directed surgical decision making for lateral pelvic lymph node dissection in rectal cancer after total neoadjuvant therapy (TNT). Ann Surg 276(4):654–664 35837891 10.1097/SLA.0000000000005589 PMC9463102

[9] Fujita S, Yamamoto S, Akasu T, Moriya Y (2009) Risk factors of lateral pelvic lymph node metastasis in advanced rectal cancer. Int J Colorectal Dis 24:1085–1090 19387660 10.1007/s00384-009-0704-4

[10] Bahadoer RR, Dijkstra EA, van Etten B, Marijnen CA, Putter H, Kranenbarg EMK, Silviera ML (2021) Short-course radiotherapy followed by chemotherapy before total mesorectal excision (TME) versus preoperative chemoradiotherapy, TME, and optional adjuvant chemotherapy in locally advanced rectal cancer (RAPIDO): a randomised, open-label, phase 3 trial. Lancet Oncol 22(1):29–42 33301740 10.1016/S1470-2045(20)30555-6

[11] Hassanzadeh C, Mirza K, Kalaghchi B, Fallahian F, Chin RI, Roy A, Kim H (2024) Lateral pelvic nodal management and patterns of failure in patients receiving short-course radiation for locally advanced rectal cancer. Dis Colon Rect 67(1):54–61 10.1097/DCR.0000000000002936 37787502

[12] Krishnatry R, Jain A, Kazi MK, Gudi S, Rane D, Pawar P, Engineer R (2024) Total neoadjuvant therapy using short course radiation with or without SIB to lateral pelvic lymph nodes in locally advanced ugly rectal cancer

[13] Garcia-Aguilar J, Patil S, Gollub MJ, Kim JK, Yuval JB, Thompson HM, Saltz LB (2022) Organ preservation in patients with rectal adenocarcinoma treated with total neoadjuvant therapy. J Clin Oncol 40(23):2546–2556 35483010 10.1200/JCO.22.00032 PMC9362876

[14] Meldolesi E, Chiloiro G, Giannini R, Menghi R, Persiani R, Corvari B, Gambacorta MA (2022) The role of simultaneous integrated boost in locally advanced rectal cancer patients with positive lateral pelvic lymph nodes. Cancers 14(7):1643 35406415 10.3390/cancers14071643 PMC8996944

[15] Chang G, Halabi WJ, Ali F (2023) Management of lateral pelvic lymph nodes in rectal cancer. J Surg Oncol 127(8):1264–1270. 10.1002/jso.27317 37222691 10.1002/jso.27317

[16] Raghavan S, Bankar S, Kazi M, Desouza A, Saklani A (2023) A novel technique for laparoscopic abdominoperineal resection with en bloc right pelvic lymph nodal dissection in the case of locally advanced low rectal cancers involving seminal vesicles—a video vignette. Colorectal Dis 25:7 10.1111/codi.16586 37165570

[17] Okamoto D, Matsuda T, Sawada R, Hasegawa H, Yamashita K, Harada H, Kakeji Y (2021) Risk factors for complications following lateral pelvic lymph node dissection for rectal cancer. Anticancer Res 41(11):5599–5604 34732431 10.21873/anticanres.15374

[18] Georgiou P, Tan E, Gouvas N, Antoniou A, Brown G, Nicholls RJ, Tekkis P (2009) Extended lymphadenectomy versus conventional surgery for rectal cancer: a meta-analysis. Lancet Oncol 10(11):1053–1062 19767239 10.1016/S1470-2045(09)70224-4

[19] Sukumar V, Kazi M, Gori J, Ankathi SK, Baheti A, Ostwal V, Saklani A (2022) Learning curve analysis for lateral pelvic lymph node dissection in rectal cancers–outcomes improve with experience. Eur J Surg Oncol 48(5):1110–1116 34893365 10.1016/j.ejso.2021.12.003

[20] Agnes A, Peacock O, Manisundaram N, Kim Y, Stanietzky N, Vikram R, Chang GJ (2024) The learning curve for robotic lateral pelvic lymph node dissection for rectal cancer: a view from the west. Dis Colon Rectum 67(10):1281–1290 38959454 10.1097/DCR.0000000000003424 PMC12324685

[21] Konishi T (2024) The use of indocyanine green for lateral lymph node dissection in rectal cancer: a novel fancy tool in the armamentarium with questionable benefits. Tech Coloproctol 28(1):1–2 10.1007/s10151-024-02964-w 39134821

